# Sympatric Atlantic puffins and razorbills show contrasting responses to adverse marine conditions during winter foraging within the North Sea

**DOI:** 10.1186/s40462-019-0174-4

**Published:** 2019-11-01

**Authors:** Katie St. John Glew, Sarah Wanless, Michael P. Harris, Francis Daunt, Kjell Einar Erikstad, Hallvard Strøm, John R. Speakman, Benjamin Kürten, Clive N. Trueman

**Affiliations:** 1 Ocean and Earth Science, University of Southampton, Waterfront Campus, Southampton, SO143ZH UK; 2 Centre for Ecology & Hydrology, Bush Estate, Penicuik, EH26 0QB UK; 3 Norwegian Institute for Nature Research, Fram Centre, N-9296 Tromsø, Norway; 4 Norwegian University of Science &Technology (NTNU), Centre for Biodiversity Dynamics, Department of Biology, N-7491 Trondheim, Norway; 5 Norwegian Polar Institute, Fram Centre, Postbox 6606, Langnes, NO-9296 Tromsø, Norway; 6 Institute of Genetics and developmental Biology, Chinese Academy of Sciences, Beijing, China; 7 Institute of Biological and Environmental Sciences, University of Aberdeen, Aberdeen, Scotland, UK; 8 School of Natural and Environmental Sciences, University of Newcastle, Newcastle-upon-Tyne, NE1 7RU UK; 9 Present address: King Abdullah University of Science and Technology (KAUST), Red Sea Research Center (RSRC), Biological and Environmental Sciences & Engineering Division (BESE), Thuwal, 23955-6900 Saudi Arabia

**Keywords:** *Fratercula arctica*, Isoscape, *Alca torda*, Marine spatial management, North Sea, Seabird foraging behaviour, Spatial ecology, Trophic ecology, Moult

## Abstract

**Background:**

Natural environments are dynamic systems with conditions varying across years. Higher trophic level consumers may respond to changes in the distribution and quality of available prey by moving to locate new resources or by switching diets. In order to persist, sympatric species with similar ecological niches may show contrasting foraging responses to changes in environmental conditions. However, in marine environments this assertion remains largely untested for highly mobile predators outside the breeding season because of the challenges of quantifying foraging location and trophic position under contrasting conditions.

**Method:**

Differences in overwinter survival rates of two populations of North Sea seabirds (Atlantic puffins (*Fratercula arctica*) and razorbills (*Alca torda*)) indicated that environmental conditions differed between 2007/08 (low survival and thus poor conditions) and 2014/15 (higher survival, favourable conditions). We used a combination of bird-borne data loggers and stable isotope analyses to test 1) whether these sympatric species showed consistent responses with respect to foraging location and trophic position to these contrasting winter conditions during periods when body and cheek feathers were being grown (moult) and 2) whether any observed changes in moult locations and diet could be related to the abundance and distribution of potential prey species of differing energetic quality.

**Results:**

Puffins and razorbills showed divergent foraging responses to contrasting winter conditions. Puffins foraging in the North Sea used broadly similar foraging locations during moult in both winters. However, puffin diet significantly differed, with a lower average trophic position in the winter characterised by lower survival rates. By contrast, razorbills’ trophic position increased in the poor survival winter and the population foraged in more distant southerly waters of the North Sea.

**Conclusions:**

Populations of North Sea puffins and razorbills showed contrasting foraging responses when environmental conditions, as indicated by overwinter survival differed. Conservation of mobile predators, many of which are in sharp decline, may benefit from dynamic spatial based management approaches focusing on behavioural changes in response to changing environmental conditions, particularly during life history stages associated with increased mortality.

## Background

How animals respond to changing environmental conditions is a key topic in movement and foraging ecology. Mobile predators may potentially respond to changes in prey availability by moving to find new resources or switching diets [1, 11, 42, 43, 45, 50, 70]. The nature of any such response reflects a balance of risk/reward associated with resource intake, energetic costs and physiological demands. The ability of sympatric species with similar ecological niches to respond to environmental variation, and the relative nature of their responses, could profoundly influence patterns of species distribution, particularly when environmental disturbances occur during critical life history stages. However, the challenge of quantifying foraging distribution and diet of sympatric species of mobile predators means that this assertion remains largely untested.

Members of the auk family (Alcidae) dominate the avian community wintering in the North Sea [59]. They forage by pursuit diving and the medium and larger sized species undergo moult of their flight and body feathers outside the breeding season (hereafter referred to as during winter). During this period of annual feather moult energy demands are high, making them potentially vulnerable to reduced prey availability and severe weather conditions [28, 55]. Like many other seabirds, annual survival rates of adult auks are typically high (*c.*0.90) with most mortality occurring in winter [28]. Ecological and environmental conditions during winter moult therefore have the potential to influence the distribution, behaviour and mortality of seabirds, but the majority of studies on environmental influences on diet and distributions of seabirds have focussed on impacts during the breeding season [30, 54, 70]. Considerably less information is available regarding changes occurring outside the breeding season.

Long-term demographic studies of three auk species breeding at the Isle of May colony in the northwestern North Sea found that annual survival rates varied synchronously in association with common environmental proxies [35]. However, the extent of population- and individual-level differences in foraging behaviours in response to different winter conditions and prey availability remain relatively unexplored. Until recently observation of behaviour during winter foraging has been impossible, but the development of bird-borne, light-based geolocators [7, 10, 18, 71] to infer foraging location, and the use of stable isotope analysis to retrospectively determine nutrient acquisition and trophic position has provided new opportunities [29, 53]. Combining stable isotope and geolocation methods reveals spatial and trophic information that cannot be retrieved from either approach in isolation, and crucially now enables us to test whether species respond to contrasting conditions during winter foraging by altering location and/or switching diet.

Models of spatial variations in the stable isotopic compositions of the pelagic food web (isoscapes) have recently been constructed for the North Sea [63]. St. John Glew et al. [58] used inferences from isoscape models, feather stable isotope data and tag data to estimate at-sea locations and diet in three species of auk (common guillemot (*Uria aalge*), razorbill (*Alca torda*) and Atlantic puffin (*Fratercula arctica*), hereafter puffin) during the winter moult period of 2014/15, a period characterised by overwinter survival rates similar to or higher than the 33 year long term average of these auk populations, and presumably favourable environmental conditions. Here we compare locational and isotopic data from puffins and razorbills during winter foraging of 2014/15 with similar data from the winter of 2007/08, when overwinter survival was markedly lower than the long-term average implying that conditions were less favourable.

Our aims were to 1) test whether differences in overwinter survival were associated with changes in locations within the North Sea and/or trophic position, 2) assess whether such responses were similar in the two species and 3) investigate whether any observed changes in locations and diet could be related to the abundance and distribution of potential prey species. For this final test we collated data on the distribution and abundance of lesser sandeel (*Ammodytes marinus*), sprat (*Sprattus sprattus*) and herring (*Clupea harengus*), lipid-rich high quality prey that are known to be common in the diet of puffins and razorbills during the breeding and winter seasons across the North Sea [26, 27, 37]. We also extracted distribution and abundance data, as well as isotope measurements for snake pipefish (*Entelurus aequoreus*), as this normally rare and nutritionally poor species showed a short-lived population explosion in 2007 and 2008, and was found in the diets of a wide range of marine predators [24, 64].

## Methods

### Survival rates

Fieldwork was carried out on the Isle of May National Nature Reserve, south-east Scotland (56°11′N, 2°34′W) where annual survival rates of adult puffins and razorbills have been estimated each year since 1984. Breeding adults were caught and marked with a numbered metal ring and a unique combination of colour rings (total sample sizes over the study: *n* = 694 puffins and 215 razorbills). In each year, visual searches were made for marked birds in the areas where they had been ringed and in other parts of the colony. The resighting data were modelled with a standard Cormack-Jolly-Seber mark-recapture model, which estimates annual survival and recapture probabilities as outlined in Freeman et al. [13], and updated to include subsequent years’ data. Permanent emigration and true mortality are confounded in the model, However, breeding adult puffins and razorbills have high colony fidelity, such that apparent survival approximates true survival [35].

### Data logger deployments

During June and July 2007 and 2014, breeding razorbills were caught using a 7 m noose pole and puffins were hand caught in their breeding burrows. Captured birds were equipped, under British Trust for Ornithology licence, with a plastic leg ring and data logger (2007: British Antarctic Survey Mark 14; 2014: Migrate Technology, UK: model w65 for puffins and c65 for razorbills; combined mass of ring and device < 0.4% body mass of both species in both study seasons). Birds were recaptured the following summer (2008 and 2015, respectively), the data loggers removed and the data retrieved. Data loggers measured light intensity at 60 s intervals and recorded the maximum value in each 10-min interval. This allowed the determination of dawn and dusk which when linked to a time base enabled the determination of latitude from the duration of night and day, and longitude from timing of local midnight or midday. Data were obtained from 10 puffins and 17 razorbills in 2008, and 12 puffins and 9 razorbills in 2015. Different individuals were sampled in the two winters. Post-processing of data logger results followed the protocols detailed in Hanssen et al. [20]. Average data logger error of individual locations has been estimated at ±186 km [47]; however, spatial accuracy was improved by removing fixes that would require unrealistic movements from adjacent locations based on visual inspection, and data collected around the equinoxes (10th September to 18th October and 20th February to 2nd April) where estimated latitudes are unreliable. Locations over land were retained, which is necessary for marine birds with coastal distributions to avoid offshore distribution bias [60]. Population kernel density maps of daily locations throughout both full winter periods were produced using the ‘bkde2D’ function in the ‘KernSmooth’ package [68] in R 3.1.2 [51]. The ‘bw.nrd’ function [57] was used to calculate the bandwidth Gaussian kernel density estimator for each species and each population. The average bandwidth value of 0.4 was used for each kernel density distribution. The difference between puffin and razorbill distribution between winters was calculated by subtracting the scaled (to lie between 0 and 1) 2007/08 kernel density surfaces from the scaled 2014/15 surface. Positive values indicate regions where more individuals were located in 2014/15, whereas negative values indicate regions where more individuals were located in 2007/08. Percentage overlap of predominant regions used by populations each year (scaled kernel density values > 0.4) were also calculated (area overlap divided by 2007/08 kernel density area).

### Stable isotope data collection and analysis

Feather samples (2–5 ventral body feathers and 2–5 feathers taken from the cheeks) were collected under UK Home Office licence from some of the recaptured birds. Hence, sample sizes for body and cheek feathers differ and no cheek feathers were obtained from razorbills in 2008 (Table 1). Feathers were stored in paper envelopes and deep-frozen until analysed.

**Table 1:** Sample sizes of birds from which geolocator data were obtained and the number of individuals, from which feathers were collected from geolocator equipped birds known to spend the winters of 2007/08 and 2014/15 within the North Sea. Timing of moult and regrowth for the Isle of May puffin and razorbill populations were taken from Harris and Wanless [22], Wernham et al., [69], Harris and Wanless, [27], Harris et al.,[28]

	Puffin 07/08		Puffin 14/15		Razorbill 07/08	Razorbill 14/15	
Number of individuals returned with GLS data	10		12		17	9	
Feather Type	Body	Cheek	Body	Cheek	Body	Body	Cheek
Isotope data + GLS data Sample Size	8	4	12	3	16	9	7
Moult Timing	Jul - Sep	Jan - Mar	Jul - Sep	Jan - Mar	Jul - Sep	Jul - Sep	Dec - Mar

Feathers were cleaned of surface contaminants using 0.25 M NaOH and rinsed with MilliQ water, oven-dried (60 °C, 12 h), then cut into small fragments avoiding the quill and shaft. A single body feather was analysed per individual, whereas cheek feathers were pooled to obtain enough material for analysis. A 0.5–0.7 mg sample was weighed into a tin capsule and bulk δ^13^C and δ^15^N values were measured. All razorbill and puffin feather samples for 2014/15 were processed at the University of Southampton and analysed by Elemtex Laboratories, Cornwall, UK on a Thermoquest EA1110 elemental analyser linked to a Sercon 2020 isotope ratio mass spectrometer. Accuracy and precision were monitored through laboratory internal standards (USGS 40 and USGS 41) and an in-house comparison standard (ARCOS glutamic acid). Accuracy was within 0.1‰ in comparison to long term averages of δ^13^C and δ^15^N measured in in-house standards, and precision was 0.1‰ for both δ^13^C and δ^15^N. Puffin feathers from 2007/08 were processed at the Centre for Ecology & Hydrology and analysed at the University of Aberdeen using an elemental analyser on the front end of a dual inlet gas source isotope ratio mass spectrometer (Micromass Ltd., Manchester, UK), but using a different set of in-house standards referenced to IAEA international reference materials (precision was 0.26‰ for δ^13^C and 0.18‰ for δ^15^N). Differences in isotopic variation between species and years were compared using ANOVA statistical tests in R 3.1.2.

Snake pipefish (subsequently referred to as pipefish) isotopic data were obtained from 62 samples opportunistically collected across the North Sea between July and November 2007 during the ICES 3rd quarter International Bottom Trawl Surveys (IBTS) on board RV *Cefas Endeavour* and in the framework of the Marine Ecosystem Connections MEC project of Cefas [34]*.* White muscle tissue samples were taken from each individual, and then samples were freeze-dried, ground and weighed (~ 1 mg) into tin capsules. The majority of stable isotope analyses were carried out using a ThermoElectron Delta XP Plus connected to a Costech ECS 4010 elemental analyser by NERC Life Sciences Mass Spectrometry Facility, East Kilbride, UK. Accuracy and precision were monitored through international standards (ammonium sulphate, USGS 25, IAEA-N1, IAEA-N2 for nitrogen and polyethylene (IAEA-CH-7), graphite (USGS 24) and sucrose for carbon). Precision was 0.3‰ for both δ^13^C and δ^15^N. A subset of pipefish samples were analysed on a ThermoElectron Delta V IRMS at Leibniz Institute for Research on Evolution and Biodiversity, FRG, using peptone as an internal standard. Precision was < 0.2‰ for both isotopes. Carbon isotope values were lipid corrected as per Kiljunen et al. [33].

Isotope data were compared to spatial statistical models (isoscapes) characterising spatial variations in the isotopic composition of carbon and nitrogen across the North Sea [63].

### Data analysis

#### Population level assignment and calibration-offset derivation

Feather stable isotope values represent nutrients assimilated in the time period immediately prior to, and during, feather growth. Therefore, the isotopic ratio is influenced by spatial variation in isotopic compositions at the base of the food web (often termed the isotopic baseline), and the taxonomic composition and trophic position of the diet that the individual was consuming during the specific period of feather moult and regrowth. Disaggregating the combined influences of spatio-temporal variation in isotopic baselines and diet is a major challenge for stable isotope analyses. Here we address this challenge using independent evidence of individual location derived from data loggers and knowledge of baseline variations drawn from isoscape models.

Winter moult timing varies between feather types and species. Population-level observational data show that body feathers of puffins and razorbills are moulted and regrown after breeding in autumn (July – September), whereas cheek feathers are grown before breeding in spring (puffins; January – March, razorbills; December–March) [22, 27, 28, 69] (Table 1). Tag-based data on temperature and immersion (wet/dry periods) has recently been used to directly identify periods when individual birds were flightless and therefore in wing moult [9, 38]. However, the tags used in our study recorded immersion but not temperature, precluding accurate classification of at-sea behaviour at the individual level. Instead our approach was to combine feather stable isotope data and light based geolocator results during the known moult time periods, to refine population location and trophic position estimates immediately prior to and during both post- and pre-breeding winter moult, without knowing the exact moult timing for each individual.

As our reference stable isotope data is limited to the North Sea [63], this investigation focuses on foraging behaviours solely within the North Sea environment and only cases where data logger points indicated that moult periods of a given feather type occurred in the North Sea were included in the subsequent analysis. Therefore, any individuals recorded outside of the North Sea area during the post- and pre-breeding moulting months listed in Table 1 were excluded from the study, resulting in the removal of feather samples from 2 puffins and 1 razorbill for 2007/08 and a cheek feather sample for a single razorbill for 2014/15. All remaining individuals stayed within the North Sea area during the moulting months assumed for each feather type.

Puffin and razorbill feather samples collected following both winters were assigned to a likely area within the North Sea using reference isoscape models [63] to estimate foraging locations immediately prior to and during both post- and pre-breeding moult. Although the isoscapes were produced from samples collected in summer 2015, the broad spatial pattern in isotopic variability across the North Sea is largely consistent over at least decadal scales, reflecting long-term stability in oceanographic and associated pelagic biogeochemical conditions conserved over time [39, 63]. Therefore, we are confident that samples collected in different years can be assigned to likely locations using the same baseline isoscape. Seabird feather assignment follows a method that has successfully assigned known-origin samples of queen scallops (*Chlamys opercularis*) and herring (*Clupea harengus*) within the North Sea [63], with scallops accurately assigned to their true origin 90% of the time when looking at an area representing 40% of the isoscape and herring assignment locations matching survey results. This same method was first used to assign seabird feathers to the North Sea by St. John Glew et al. [58].

**Fig. 1:**
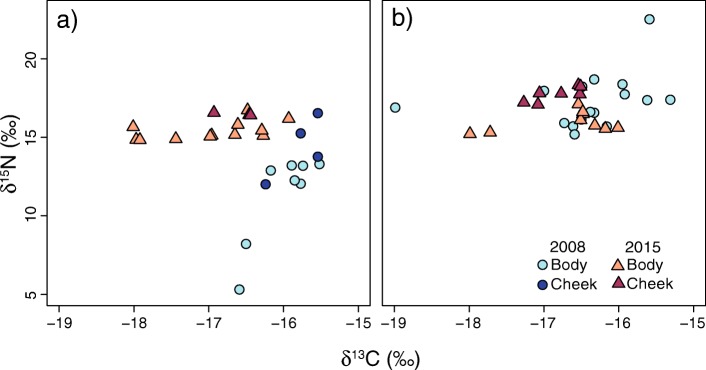
Biplots of δ^13^C and δ^15^N isotope values of puffin (**a**) and razorbill (**b**) feathers grown in the winters of 2007/2008 (poor survival year; circles) and 2014/2015 (high survival year; triangles)

The North Sea isoscapes were produced using lion’s mane jellyfish (*Cyanea capillata*) as a reference organism, to a resolution of 0.1 degrees [39, 63]. To carry out the assignment of seabird feathers to the likely foraging location during moult, a calibration was required to account for differences between the isotopic compositions of reference jellyfish tissues and those of seabird feathers resulting from different amino acid compositions between the sampled tissues and trophic level differences. The degree of isotopic offset (calibration – offset) between jellyfish and bird feathers was estimated at the population-scale (i.e. a median value for all individuals within a population) by aligning North Sea isoscapes and data-logger derived population kernel density areas, i.e. independent location estimates, for both puffin and razorbill populations for each feather type in both years as per Fig. 1 in St. John Glew et al. [58]. Briefly, coordinates from population kernel density areas (with density values greater than 0.01), representing all of the locations within the North Sea visited by the birds during the assumed feather specific seasonal moult periods (Table 1), were recovered and the δ^13^C and δ^15^N values associated with these locations were extracted from the δ^13^C and δ^15^N isoscapes. Population median and standard deviation isoscape δ^13^C and δ^15^N values were then calculated. The population calibration-offset and associated standard deviations were derived as the average isotopic difference between 1000 random draws of a normal distribution based on the median and standard deviation values within the isoscape area and the median and standard deviation values measured in the population of feathers (Fig. 1; St. John Glew et al. [58]). Isotopic differences associated with the difference in protein compositions between jellyfish and feather proteins were assumed to be constant within species and feather type. Thus, any remaining differences in calibration-offset values between species and feather types were assumed to represent isotopic differences in diet, and were statistically compared within species between years. We assume that any remaining physiological differences potentially influencing isotopic compositions such as differences in dietary protein quality or physiological stress [40] are relatively minor compared to effects of foraging at different trophic levels, at least at the population level.

We estimated the most likely feeding areas during both post- and pre-breeding winter moult at the population level using the methodology described in Trueman et al. [63] and St. John Glew et al. [58] with the addition of a Bayesian framework to include prior information from data logger-derived location estimates (methodology for Bayesian isoscape assignments using priors are described by Wunder [72] and Vander Zanden et al. [65]). Feather isotope data were matched to the jellyfish isoscapes using the derived calibration-offset and combined variance values described above (the assignment conditions are summarised in Table 2). Likely feeding locations were identified by estimating the likelihood that each raster cell of the North Sea carbon and nitrogen isoscapes represented the foraging area of each individual, using the bivariate normal probability function and prior knowledge of known winter locations using the population kernel density areas. Probability raster surfaces derived from data loggers were treated as prior probabilities of location during the moult period and posterior probability density surface was defined as the product of the prior and isotope assignment probability raster surfaces. Based on accuracy and precision sensitivity analyses presented in Trueman et al. [63], all cells with probabilities exceeding a defined threshold likelihood of 1.42; which represents the highest 30% of the assignment probabilities, were considered likely areas.

**Table 2:** Assignment conditions adopted for stable isotope-based location of puffins and razorbills against isoscapes derived from jellyfish tissue [58]

Variable	Isoscape Jellyfish	Seabird Assignment			
		Puffin		Razorbill	
		2007/08	2014/15	2007/08	2014/15
Measurement error (σ)	δ^13^C & δ^15^N: 0.2	δ^13^C & δ^15^N: 0.2			
Between-individual variance (measured)	δ^13^C: 0.78, δ^15^N: 1.02	δ^13^C: 0.60, δ^15^N: 2.74	δ^13^C: 0.52, δ^15^N: 0.91	δ^13^C: 0.63, δ^15^N: 2.10	δ^13^C: 0.51, δ^15^N: 0.58
Calibration-Offset and variance values	NA	*Derived – see Results* Table 3 *below.*			
Threshold odds ratio	NA	1.42			

Foraging locations were compared between years by overlaying assignment surfaces and calculating percentage overlap of likely foraging areas. Species and feather type Bayesian probability assignment surfaces were mapped in R 3.1.2 [51].

#### Prey abundance

Data on the distribution and abundance of high-quality prey items (sandeels, sprat and herring) were extracted from North Sea IBTS data ([32], https://datras.ices.dk). Hourly catch per unit effort data (CPUE) for each ICES statistical rectangle (30 min latitude by 1° longitude), were extracted for herring, sprat and sandeel that were small enough to be eaten by these seabirds (defined as individuals < 160 mm [31], < 80 mm [31], and < 120 mm [4], respectively). Data were obtained for January to March 2008 and 2015. Prey abundance data were not available for October to December 2007 or 2014. CPUE data were averaged for each ICES rectangle and displayed as log10(CPUE/hr. + 1) to graphically display differences in abundance across orders of magnitude. CPUE for each species was statistically compared between the two study years within the northern North Sea (> 55°N). Snake pipefish CPUE data were obtained for each ICES statistical rectangle for January to March in 2008 and 2015, and statistically compared between years across the entire North Sea.

#### Snake pipefish isotopic variability

Estimates of likely spatial variations in the isotopic compositions of pipefish across the wider North Sea were produced from the measured isotopic composition of snake pipefish muscle tissue from known origin individuals, extrapolated across the region using ordinary kriging in R 3.1.2 [51] (Additional file 1: Figure S1). Estimated pipefish δ^13^C and δ^15^N values were extracted for coordinates matching the most likely feeding areas as estimated from combined geolocator and isotope results. Coordinates of likely foraging location during cheek and body feather regrowth were combined, and the mean extracted isotope values calculated for each species, as pipefish samples were collected across the whole of the birds’ non-breeding period and likely foraging locations were consistent between feather types during 2007/08 moult. Muscle samples of sprat, herring and sandeels were not collected in either winter period, therefore stable isotope compositions for these species were lacking.

## Results

### Adult survival

Survival rates of adult puffins and razorbills breeding on the Isle of May are normally high (means of 33 years; puffin 0.921 (95% Credible Interval 0.900–0.939); razorbill 0.923 (0.897–0.945)), but survival over the 2007/08 winter was much reduced (puffin 0.721 (0.643–0.791); razorbill 0.828 (0704–0.923); 100 and 96.9% of the posterior distribution below the long-term average, respectively). In contrast, over the 2014/15 winter, the survival of puffins was higher than average (0.945 (0.904–0.977; 11.2% of the posterior below the long term average) and that of razorbills was average (0.894 (0.788–0.964); 67.2%).

### Stable isotope results

Population median nitrogen and carbon isotope values of both feather types of puffins (Mann Whitney U tests: Nitrogen; W = 20, *p* < 0.05, Carbon; W = 166, *P* < 0.05) and nitrogen isotope values of razorbills (W = 113.3, *P* < 0.05) differed significantly between 2014/15 and 2007/08 (Fig. 1, Table 3). Razorbill carbon isotope values did not differ significantly between years. Median carbon and nitrogen isotope values for puffin body feathers were significantly lower in 2007/08, when survival rates were poor, compared to 2014/15 (Carbon: W = 87, *P* < 0.05, Nitrogen; W = 0, *P* < 0.05). During 2007/08, nitrogen isotope values of puffin cheek feathers were similar to those from 2014/15, whereas carbon isotopic values of cheek feathers differed significantly between years (W = 12, *P* < 0.05). Median body feather carbon isotope values for razorbill were similar between years, but median δ^15^N values in razorbill body feathers were significantly higher in 2007/08 (W = 113.5, *P* < 0.05) compared to 2014/15 (Table 3).

**Table 3:** Population median and standard deviation δ^13^C and δ^15^N feather values of puffins and razorbills and the population calibration-offset values and standard deviations calculated from the difference between median isoscape extracted isotope values within the population kernel density areas for each feather type and median measured feather isotope values of razorbills and puffins in winters 2007/8 and 2014/15

Feather Type	Puffin 2007/08		Puffin 2014/15		Razorbill 2007/08	Razorbill 2014/15	
	Body	Cheek	Body	Cheek	Body	Body	Cheek
δ^13^C: Median & (σ)	−15.87 (0.38)	−15.65 (0.33)	−16.81 (0.72)	−16.46 (0.27)	− 16.47 (0.71)	−16.48 (0.68)	− 16.77 (0.31)
δ^15^N: Median & (σ)	12.58 (2.94)	14.52 (1.95)	15.15 (0.58)	16.43 (0.09)	16.44 (1.40)	17.79 (0.46)	15.76 (0.65)
Δ^13^C_f-j_: Cal. offset & (σ)	−0.02 (0.71)	0.57 (0.95)	−0.60 (1.03)	−0.21 (0.99)	0.65 (1.45)	1.11 (0.86)	−0.07 (1.13)
Δ^15^N_f-j_: Cal. offset & (σ)	1.74 (2.99)	4.29 (2.19)	4.63 (0.92)	6.02 (0.99)	6.13 (2.00)	6.30 (1.73)	4.78 (1.22)

Isotopic variability within populations and feather types differed between years. In the winter with average to high survival rates (2014/15), within population isotope variability was relatively constant between species with δ^15^N and δ^13^C standard deviations ranging from 0.09–0.58‰ and 0.27–0.72‰ respectively across feather types in puffins, and 0.46–0.65‰ and 0.31–0.68‰ in razorbills. In the winter of 2007/08, when survival rates were lower, carbon isotope variability in puffin feathers (0.33–0.38‰) was similar to razorbill feathers (0.71‰) and between sample years, but variability in δ^15^N values was higher in both species (1.59–2.94‰ in puffins and 1.40‰ in razorbills). Year had a significant effect on δ^15^N variability (ANOVA: F = 18.83, *P* < 0.001), whereas no significant year effect was apparent in δ^13^C variability in either species (ANOVA: F = 0.09, *P* > 0.05). Feather type and species had no significant effect on isotope values. All isotope data are displayed in Fig. 1, summary data are provided in Table 3.

### Wintering and moulting locations

**Fig. 2:**
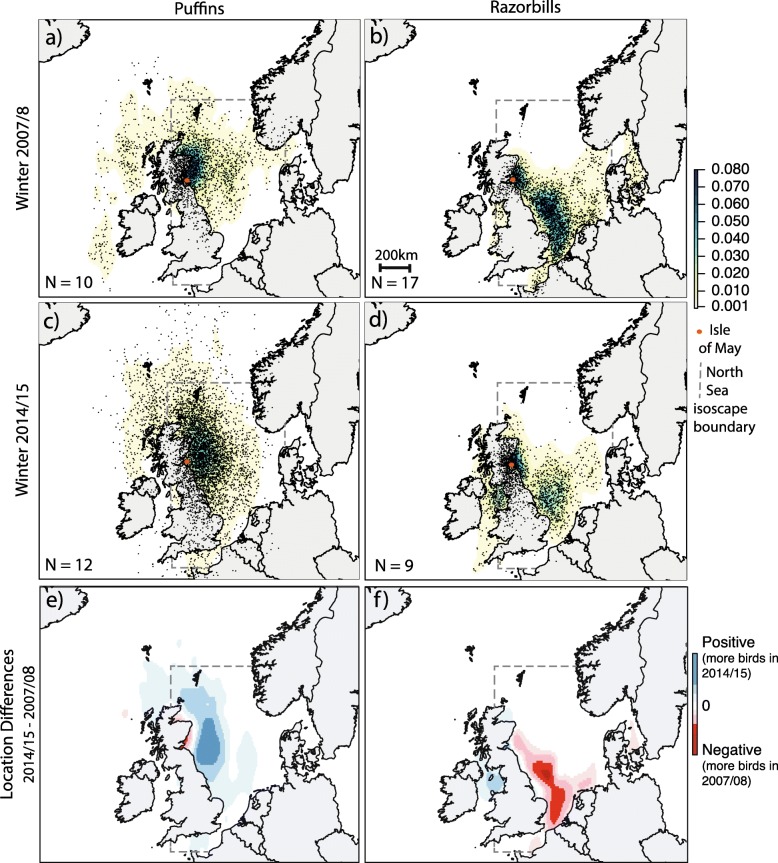
Kernel density surface depicting population spatial usage around the UK using coordinate data collected from light-based geolocators attached to populations of puffins (**a** = 2007/08, **c** = 2014/15) and razorbills (**b** = 2007/08, **d** = 2014/15) during the entire non-breeding period (Jul-March). Kernel is calculated as the standard bivariate normal density, with higher values representing greater use regions. Individual data points are also overlaid. All individuals for which geolocator data was obtained were included. The difference (2014/15–2007/08) between the scaled kernel density surfaces for both puffins (**e**) and razorbills (**f**) are also displayed. Positive values (blue) indicate regions where more individuals were located in the winter of 2014/15, negative values (red) indicate regions where more individuals were located in the winter of 2007/08

Results from the data loggers showed that during both winters puffins were located mainly in the northwest North Sea, with an 89% overlap in predominant foraging locations between winters. The highest kernel densities were within approximately 400 km of the Isle of May (Fig. 2a,c,e). In both winters some puffins left the North Sea (6/10 individuals in 2007/08 and 6/14 individuals in 2014/15) but the frequency did not differ significantly between years (Chi-squared analysis: Χ^2^ = 0.17, *P* = 0.67). Individual razorbills were also recorded leaving the North Sea in both years (3/17 individuals in 2007/08 and 5/9 individuals in 2014/15), and again there was no difference in frequency between years (Chi-squared analysis: Χ^2^ = 2.38, *P* = 0.12). In contrast to the situation in puffins, where the population remained in a broadly similar region (northwestern North Sea) between years, data logger-defined wintering areas used by razorbills differed between winters (Fig. 2b,d,f), with only a 16% overlap in predominant foraging locations between winters. In 2007/08, birds were predominantly located in the southern North Sea (with kernel density values of 0.04–0.06), whereas in 2014/15 the majority of birds used areas off southeast and northeast Scotland and within the Firth of Forth, with less use being made of the southern North Sea (with kernel density values of 0.01–0.03). The highest kernel densities for razorbills in the winter of 2014/15 were within approximately 120 km of the Isle of May (Fig. 2).

**Fig. 3:**
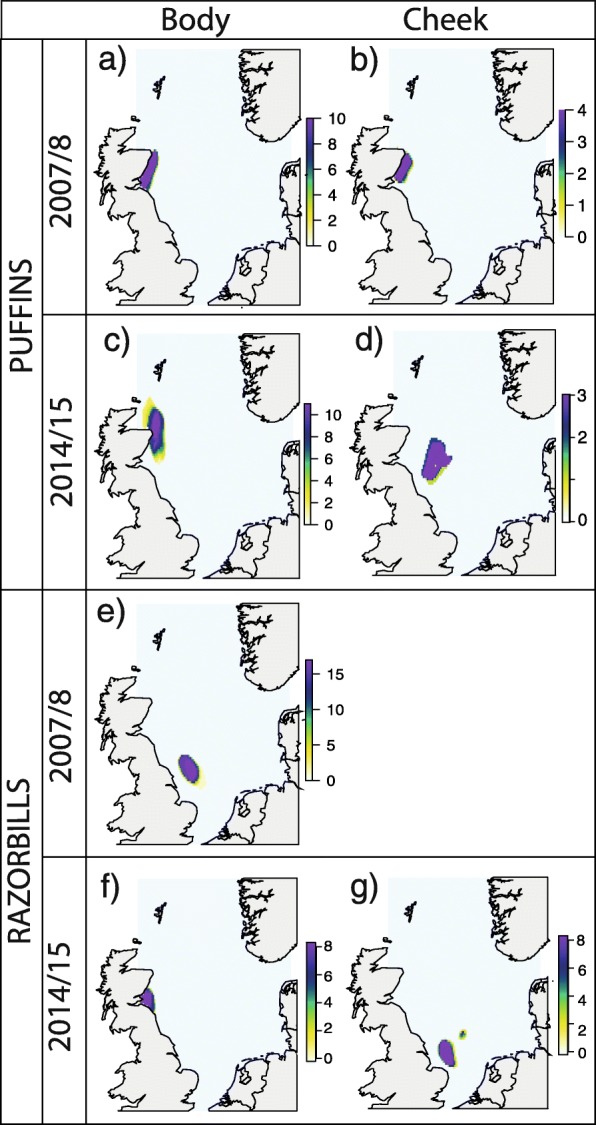
Bayesian probability assignments using derived calibration-offset and season-specific kernel density areas as prior probability surfaces of puffin (**a**, **b**, **c**, **d**) and razorbill (**e**, **f**, **g**) body and cheek feathers collected in winter 2007/08 (**a**, **b**, **e**) and 2014/15 (**c**, **d**, **f**, **g**). Regions identified represent the most likely foraging regions during moult

In 2007/08, data loggers indicated that 8 out of 10 puffins were in the North Sea during the months when both body and cheek feather moult occurred (Table 1) and could therefore be assigned to the North Sea isoscape to infer post- and pre-breeding moult locations. In 2014/15, moult locations could be estimated for all individuals. As with the overall wintering distribution, regions identified as likely areas within the North Sea used by puffins for both post- and pre-breeding moulting were broadly similar in the two winters (Fig. 3a-d). During winter 2007/08 body and cheek feather moult most likely occurred in the northwestern North Sea, close to the Isle of May (Fig. 3a-b). In 2014/15 both post- and pre-breeding moult again occurred in the northwestern North Sea. However, post-breeding body moult and particularly pre-breeding cheek moult occurred further offshore in 2014/15 compared to 2007/08 (Fig. 3c-d). Thus, foraging regions during body feather moult showed a 64% overlap in the two winters, while cheek feather moulting locations did not overlap.

Most razorbill individuals remained within the North Sea during known post- and pre-breeding moulting months (Table 1) (94% in 2007/08 and 88% in 2014/15) and were therefore assigned to the North Sea isoscape. The most likely post-breeding moult locations differed between the two studied winters (Fig. 3e-g). Thus, body feather growth in 2014/15 most likely occurred in the northwestern North Sea, close to the Isle of May, whereas body feather growth in 2007/08 occurred in the central North Sea, in a similar region to cheek feather growth in 2014/15.

### Trophic position

Changes in trophic position are revealed by differences in the isotopic offset between bird feathers and the jellyfish isoscape (Table 3). In 2014/15, the range in puffin nitrogen offset values (Δ^15^Ν_f-j_) was 4.63–6.02‰, similar to that of razorbills (4.78–6.30‰,). However, in 2007/08 puffin nitrogen offset (Δ^15^Ν_f-j_) was significantly lower than in 2014–15 in the period prior to and during post-breeding body feather moult (1.74‰ higher than jellyfish) (Mann Whitney U test: W = 832,865, *P* < 0.05) (Table 3). In contrast, during 2007/08 razorbill nitrogen offset (Δ^15^Ν_f-j_) values were significantly higher in the period prior to and during post-breeding body feather moult (6.13‰ higher than jellyfish) (Mann Whitney U test between years: W = 280,242, *P* < 0.05) (Table 3). In both species and across all feather types, isotopic variance was increased in the winter of lower survival rates (Table 3).

### Prey availability and likely prey items

**Fig. 4:**
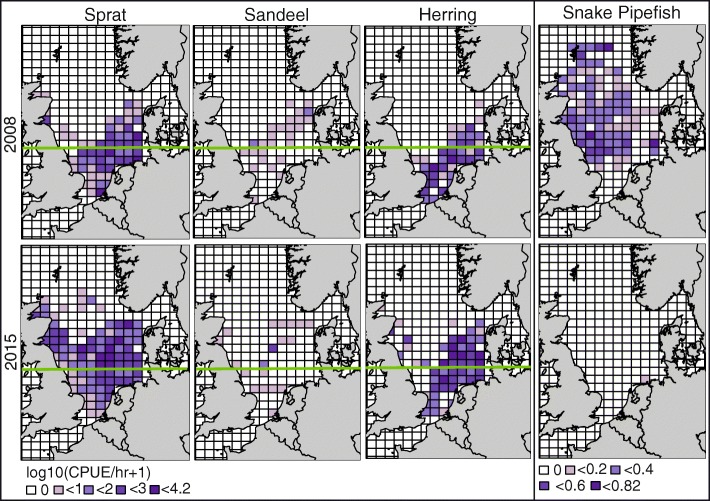
Catch per unit effort (CPUE) per hour data, per ICES statistical rectangle for age class 0 sprat, sandeel, herring and snake pipefish, in quarter 1 in 2008 and 2015. CPUE/hr. values were averaged in each ICES rectangle and displayed as log10(CPUE/hr. + 1). Data were obtained from ICES North Sea IBTS database. Green line indicates the 55° mark, separating the northern and southern North Sea

The abundance and spatial distributions of sprat, sandeel and herring differed between the two winters. In 2014/15, CPUE of herring and sprat were significantly higher in the northern (> 55°N, North of the Dogger Bank which divides the southern and northern North Sea) North Sea (Mann Whitney U tests: herring; W = 5177, *P* < 0.05, sprat; W = 5882, *P* < 0.05) compared to 2007/08. Sandeel CPUE did not significantly differ between years (W = 749, *P* > 0.05). All three species had a more northerly distribution compared to 2007/08, when prey populations were largely concentrated within the southern North Sea (Fig. 4). Pipefish CPUE was high in 2007/08 and the species widely distributed across the North Sea. By contrast, CPUE was significantly lower in 2014/15 (W = 13,208, *P* < 0.05) and pipefish distribution was limited (Fig. 4). The isotopic difference between pipefish muscle tissue and puffin and razorbill feather tissues in the most likely moulting regions was 2.46‰ and 1.65‰ for carbon and 3.67‰ and 6.81‰ for nitrogen, respectively (Table 4). Assuming likely isotopic spacing of c3.5‰ in δ^15^N values between predators and prey [63], these data are consistent with pipefish forming a contribution to puffin diet, but not razorbill diet in 2007/8.

**Table 4:** Average puffin and razorbill feather carbon and nitrogen isotope values across all feather type samples collected in winter 2007/8, average pipefish isotope values extracted from pipefish carbon and nitrogen isoscapes, produced from samples collected in winter 2007/8, within the highest likely foraging regions during moult of both puffins and razorbills, and the difference between these values

	Puffin		Razorbill	
	Carbon	Nitrogen	Carbon	Nitrogen
Average feather isotope values (‰)	−15.76	13.55	−16.47	16.77
Average pipefish isotope values in most likely foraging locations during feather moult (‰)	−18.22	9.88	−18.12	9.96
Difference between pipefish isotope values and seabird isotope values (‰)	2.46	3.67	1.65	6.81

## Discussion

By combining geolocator data with stable isotope data recovered from feathers grown during winter moult and isoscape models, we compared location and trophic level immediately prior to and during at-sea post- and pre-breeding winter moult in two sympatric auk species across two winters of contrasting mortality levels. Overwinter survival of puffins and razorbills from the Isle of May was lower in 2007/08 (by 0.721 and 0.828) compared to 2014/15 (0.945 and 0.894) and survival of a third auk species, the common guillemot at this colony was also depressed in 2007/08 [35]. Such variations in survival are likely to be associated with different environmental conditions, particularly prey availability outside the breeding season [5, 55]. Our data logger and isotopic data for puffins and razorbills from the Isle of May indicate that these populations showed contrasting responses to under different winter conditions. Puffins foraged in broadly similar areas during both winters but fed at a lower trophic level during post-breeding moult in the year when survival was poor, whereas razorbills foraged at a higher dietary trophic level and also altered foraging area, having a more southerly (distant) distribution during post-breeding body feather moult when survival was poor.

Lahoz-Monfort et al. [35] speculated that synchrony in survival rates of Isle of May auk populations were associated with shared wintering areas and/or shared prey species, influenced by changing environmental conditions. The abundance and distribution of potential prey species of puffins and razorbills suggest that conditions in the two winters did indeed differ. Thus in 2007/08 when auk survival rates were low, the abundance of high-quality prey species such as lesser sandeel, herring and sprat was lower in the waters around the Isle of May and prey were mainly located in the southern North Sea. The 2007/08 winter was also characterised by an unusually high abundance and widespread distribution of snake pipefish which are of low nutritional value and difficult for small to medium sized seabirds like puffins and razorbills to handle and digest [21, 64]. The locational and trophic level data for puffins and razorbills are consistent with these changes in prey abundance and distribution. Thus in 2007/08 puffins remained in the northwestern North Sea where high quality prey were largely absent resulting in consumption of lower quality available prey, and isotopic data were consistent with the inclusion of pipefish into the diet. Razorbills by contrast, migrated to more distant wintering areas in the southern North Sea, where they were able to obtain higher trophic level diets with no evidence that snake pipefish were eaten. Previous studies have reported shifting summer foraging areas in response to changing prey availability in razorbills [15, 61], whilst puffins have been recorded as maintaining overwintering locations despite changed environmental conditions [25]. Puffins are also known to lower their trophic position and adopt more opportunistic foraging strategies during the harsher winter months [26, 29].

Foraging flexibility is important for survival when prey abundance and distribution varies [19], and recent evidence supports the theory that reduced foraging site fidelity may be ecologically advantageous in variable climatic conditions [1]. However, a trade-off is likely to exist between the increased energy expenditure associated with longer migration costs compared to maintaining local foraging and adapting to a lower trophic level (and potentially lower quality) diet [6, 25]. Additional information on foraging efficiency and individual level field metabolic rates would help tease apart these differential behavioural responses and clarify the implications for population survival between years.

Crucially, the two sympatric species studied here showed divergent responses to contrasting winter conditions over two distinct years. Divergent foraging behaviours were primarily observed during post-breeding moult, where puffins were found to maintain a broadly similar foraging location between years, but reduced their trophic position in the poor survival year, whereas razorbills shifted foraging location and increased their trophic position in the poor survival year. In the pre-breeding moult, puffins foraged at similar trophic levels across both years. We did not have samples reflecting pre-breeding moult for razorbills in 2007/08. Differences in foraging behaviours after breeding could be due to the increased and different levels of stress experienced by both species during breeding, subsequently influencing post-breeding foraging activities [56]. Alternatively, contrasting foraging behaviours during post-breeding moult may be due to differences in feathers moulted at this time. Razorbills moult both cheek and secondary feathers post-breeding, and are flightless, therefore perhaps requiring individuals to seek out high density prey areas prior to this time. On the other hand, secondary feather moult does not occur alongside cheek feather moult in puffins, perhaps reducing the need to migrate to alternative prey rich areas prior to moult.

Ecological theory suggests that niche partitioning can be advantageous when multiple species are competing for similar resources [48]. Increased diet specialization or habitat selection is likely to occur when competition is most prevalent; e.g. during times of limited prey availability or prey quality [8, 48]. Foraging niche divergence may enable sympatric species to avoid interspecific competition and enhance their ability to persist, particularly when prey resources are scarce [2, 48]. However, survival of puffins in the winter of 2007/08 was depressed to a greater extent compared to that of razorbills implying that for this winter at least, the strategy pursued by razorbills was more successful. With ever increasing unpredictability of future climates and the likely impacts on prey availability, the success of species’ responses will be central in their future resilience to environmental change.

Although we demonstrate differences in winter resource use within the North Sea by razorbills and puffins particularly during post-breeding moult, relatively few individuals were sampled and high individual variability was observed. As the same individuals were not sampled between years, it is possible that the contrasting behaviours observed here are simply a result of individual foraging differences within each population. However, isoscape assignments were performed on all individuals and summed together as a population, and very limited foraging location variations were observed among individuals from both populations (Fig. 3). It is therefore parsimonious to suggest that the observations do reflect population-level shifts in foraging location between the two studied years rather than consistent individual-level differences in behaviour and sampling that, by chance, selected individual puffins with contrasting tropic behaviours and individual razorbills with contrasting spatial behaviours between years. If foraging location differences were a result of individual differences alone, we would also expect a larger variation in assignment location across each population.

Our study also focussed on a single colony and was restricted to comparing foraging behaviours within the North Sea environment. Our results cannot, therefore, be extrapolated to infer adaptive responses in populations from other breeding colonies in regions outside the North Sea. In addition there are methodological limitations due to sampling feasibility, as outlined by St. John Glew et al. [58]. Additional isotope measurements of the high-quality prey species (sprat, herring and sandeels) would provide a clearer indication of diet composition between years, although comparison of population trophic levels undoubtedly indicates that puffin diets did differ between years even though the actual species consumed is unknown. We also appreciate that food availability is only one of many factors that may have influenced population survival rates, such as water temperatures, wind conditions and presence of anthropogenic pressures. However, from the results presented here we can conclude that a marked change in prey abundance and distribution did coincide with a change in auk foraging behaviours and in differential survival rates between years.

Our findings are potentially relevant to the spatial conservation management of mobile predators in the North Sea. Seabirds in many regions are experiencing combined adverse effects of reduced prey availability and increased offshore development for the renewable energy industry [12, 14, 16, 17, 23]. Research into the ability of seabird populations to adapt their foraging behaviours is therefore critical to providing effective long-term conservation strategies that are robust to changing future climates and increased anthropogenic pressures. We are now able to identify important seabird foraging areas within the North Sea during the breeding season [67]. During this period the areas individuals can exploit are constrained by the need for members of a pair to return regularly to the breeding site to incubate the egg and provision the chick. Foraging ranges vary markedly among species from tens to hundreds of kilometres as a result of differences in life history characteristics and foraging strategies [62]. However, outside the breeding season individuals migrate away from the colony enabling central place foraging constraints to be relaxed and mobility to increase. The establishment of Marine Protected Areas (MPAs) is considered fundamental to future seabird conservation and there is an urgent need to better define at-sea foraging locations and spatial ecology of seabirds, so appropriate and effective management areas can be designated [36, 41, 46, 66]. To date MPA designation has largely been focussed on areas that are important during the breeding season [3, 49]. There is increasing recognition that such designations should capture the interannual variation in distribution associated with fluctuations in environmental conditions, to ensure that MPAs provide robust, long-term protection [3]. New efforts are underway to protect seabirds outside the breeding season [44, 52], and our findings emphasise the importance of incorporating interannual variation in distribution and resource use at this time. At least in some species, area use may vary with contrasting conditions. Some areas may only be used when conditions are less favourable, yet protecting these areas could be critical for enabling a population to survive when conditions are poor. Protecting the areas used by species such as razorbills that adjust location with conditions is a considerable challenge, because of the large areas involved. One management option to address this challenge is the development of dynamic MPAs, whereby locations and boundaries are changed in response to season, local environmental conditions and predicted behavioural responses of mobile predator populations.

## Conclusions

The combination of light based data logger geolocation and stable isotope assignment techniques can be an effective tool to study combined spatial and trophic aspects of foraging strategies and adaptations to contrasting winter conditions in mobile populations living in remote environments. There is also empirical support for the assertion that sympatric, ecologically similar species persist by showing opposing responses to differences in local conditions. These findings also suggest that spatial conservation management must consider the dynamic responses of mobile species to variation to environmental conditions.

## Supplementary information

**Additional file 1: Figure S1.** Carbon (A) and nitrogen (B) isoscape models based on pipefish tissue samples. Sample locations are indicated by filled circles.

## Data Availability

All isotope data and code files are available in the associated data repository: github.com/katiestjohnglew/UKSeabird2018b.
