# Supplementary figures and images for: Sympatric Atlantic puffins and razorbills show contrasting responses to adverse marine conditions during winter foraging within the North Sea
Source: Mov Ecol. 2019 Nov 1;7:33. doi: 10.1186/s40462-019-0174-4 (PMC6824136; doi:10.1186/s40462-019-0174-4)

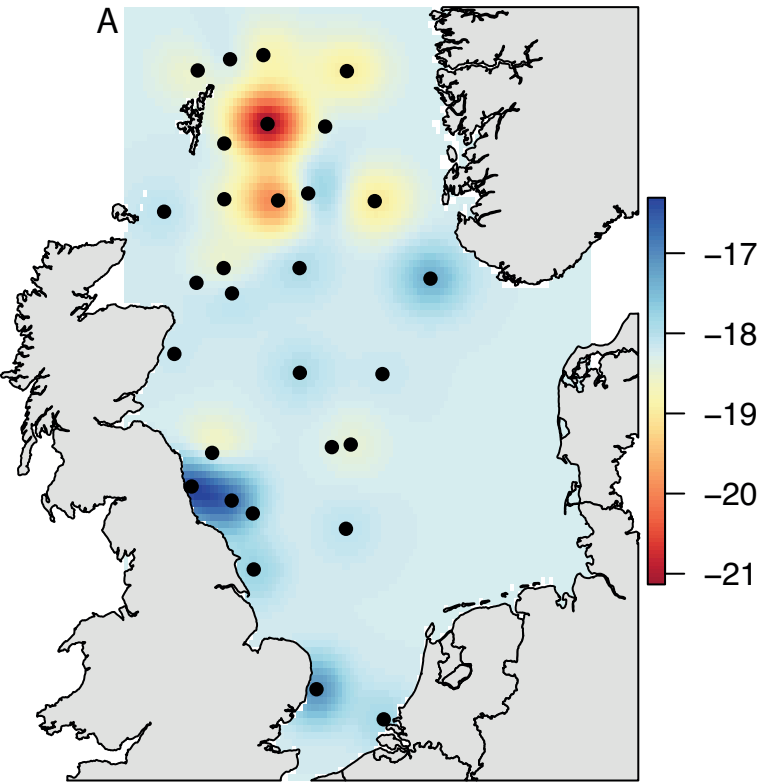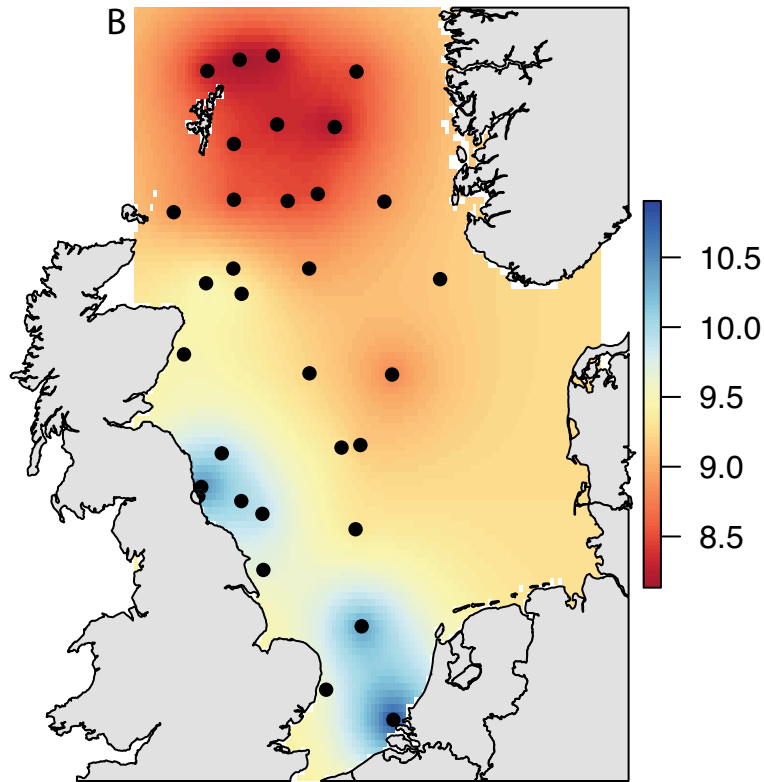

Supplement: Supplementary file 1 — Additional file 1: Figure S1. Carbon (A) and nitrogen (B) isoscape models based on pipefish tissue samples. Sample locations are indicated by filled circles. [file 40462_2019_174_MOESM1_ESM.pdf]
